# Self-Trained Convolutional Neural Network (CNN) for Tuberculosis Diagnosis in Medical Imaging

**DOI:** 10.7759/cureus.63356

**Published:** 2024-06-28

**Authors:** Karan Sarawagi, Ashutosh Pagrotra, Hardik Dhiman, Navjot Singh

**Affiliations:** 1 Department of Computer Science, Chandigarh University, Mohali, IND

**Keywords:** chest x-ray, image processing, diagnostic accuracy, medical imaging, deep learning, convolutional neural network, tuberculosis

## Abstract

Background

Tuberculosis (TB) is a serious infectious disease that primarily affects the lungs. Despite advancements in the medical industry, TB remains a significant global health challenge. Early and accurate detection of TB is crucial for effective treatment and reducing transmission. This article presents a deep learning approach using convolutional neural networks (CNNs) to improve TB detection in chest X-ray images.

Methods

For the dataset, we collected 7000 images from Kaggle.com, of which 3500 exhibit tuberculosis evidence and the remaining 3500 are normal. Preprocessing techniques such as wavelet transformation, contrast-limited adaptive histogram equalisation (CLAHE), and gamma correction were applied to enhance the image quality. Random flipping, random rotation, random resizing, and random rescaling were among the techniques employed to increase dataset variability and model robustness. Convolutional, max-pooling, flatten, and dense layers comprised the CNN model architecture. For binary classification, sigmoid activation was utilised in the output layer and rectified linear unit (ReLU) activation in the input and hidden layers.

Results

The CNN model achieved an accuracy of ~96.57% in detecting TB from chest X-ray images, demonstrating the effectiveness of deep learning, particularly CNNs, in this application. Self-trained CNNs have optimised the results as compared to the transfer learning of various pre-trained models.

Conclusion

This study shows how well deep learning-in particular, CNNs-performs in the identification of tuberculosis. Subsequent efforts have to give precedence to optimising the model by obtaining more extensive datasets from the local hospitals and localities, which are vulnerable to TB, and stress the possibility of augmenting diagnostic knowledge in medical imaging via machine learning methodologies.

## Introduction

Despite advancements in the medical industry, tuberculosis (TB) remains a significant global health challenge, infecting more than ~10.7 million new people and resulting in ~1.3 million deaths, as per the report of the WHO in 2023 alone. TB also ranks second on the list of infectious killers after the COVID-19 pandemic [1]. Effective TB therapy and disease control depend on early and precise diagnosis, particularly in impoverished areas where access to cutting-edge medical facilities is limited. The first technique to identify tuberculosis (TB) was the tuberculin skin test, followed by more recent diagnostic techniques such as sputum microscopy, chest X-rays, and molecular testing [2]. These traditional techniques are not up to the standard that is needed for clinical trials and come with a bunch of drawbacks like the requirement of skilled staff.

To reduce the cost of the TB screening process, mainly chest X-rays (CXRs) are used for diagnosis but they contain a major problem of internal sensitivity which comes due to malfunctioning of the X-ray machine. In order to solve these problems, computer-aided detection (CAD) techniques came into the picture, which laid emphasis on the integration of convolution-based deep learning systems [3]. Radiologists and medical physicians use this technique hassle-free without the use of any sophisticated or complicated equipment.

Deep learning models are highly advanced networked systems and are a subset of artificial intelligence. They can create various classification systems that can easily identify various recurring patterns and features from large amounts of data provided by various organizations. Since they studied extensive information on chest X-ray images, they used some algorithms to help identify various abnormalities that could accurately indicate TB symptoms.

Convolutional neural networks (CNN) have a great impact on medical image processing [4]. They are very effective in image classification and are an important part of deep learning. CNN examines each pixel in the chest X-ray by first dividing it into multiple matrices. They then used methods such as random selection and grid search to find specific patterns, such as nodules. CNNs can be trained to extend their learning models to accurately classify tuberculosis cases, even when symptoms are mild or unusual. This technical advancement makes TB screening processes more accessible and ubiquitous in remote areas. Also, it is duly easier as compared to the previous hectic processes without machines, especially where qualified radiologists are limited.

## Materials and methods

Dataset description

We collected a variety of chest X-rays from reputable sources. Our main source of data was a well-known website named Kaggle.com [5]. Focusing mainly on four datasets: the Montgomery and Shenzhen databases, which come under the Medicine Library of the US; the Belarus dataset, which was gathered in the past for research on drug addiction by the Belarus Ministry of Health; the National Institute of Allergy and Infectious Diseases (NIAID) TB dataset; and the Radiological Society of North America (RSNA) dataset, which was used previously for pneumonia identification, containing vast amounts of normal and TB-labelled images. The medical imaging community makes extensive use of these databases for research. We sent more than 35,000 images for screening, making our dataset very large. This combined dataset's diversity made it ideal for us to train and validate our model for tuberculosis diagnosis.

**Figure 1:**
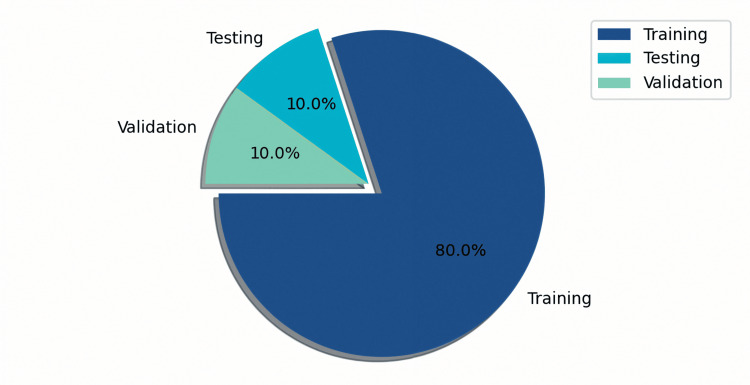
Visual representation of dataset categorised into training, testing and validation

Figure 1 shows that the dataset was divided into an 80% training set, and the remaining 20% was equally divided into a 10%-10% validation and testing set, following normative machine learning practices. An image generator was used to shuffle the dataset and generate the training, validation, and testing datasets.

Data pre-processing

**Figure 2:**
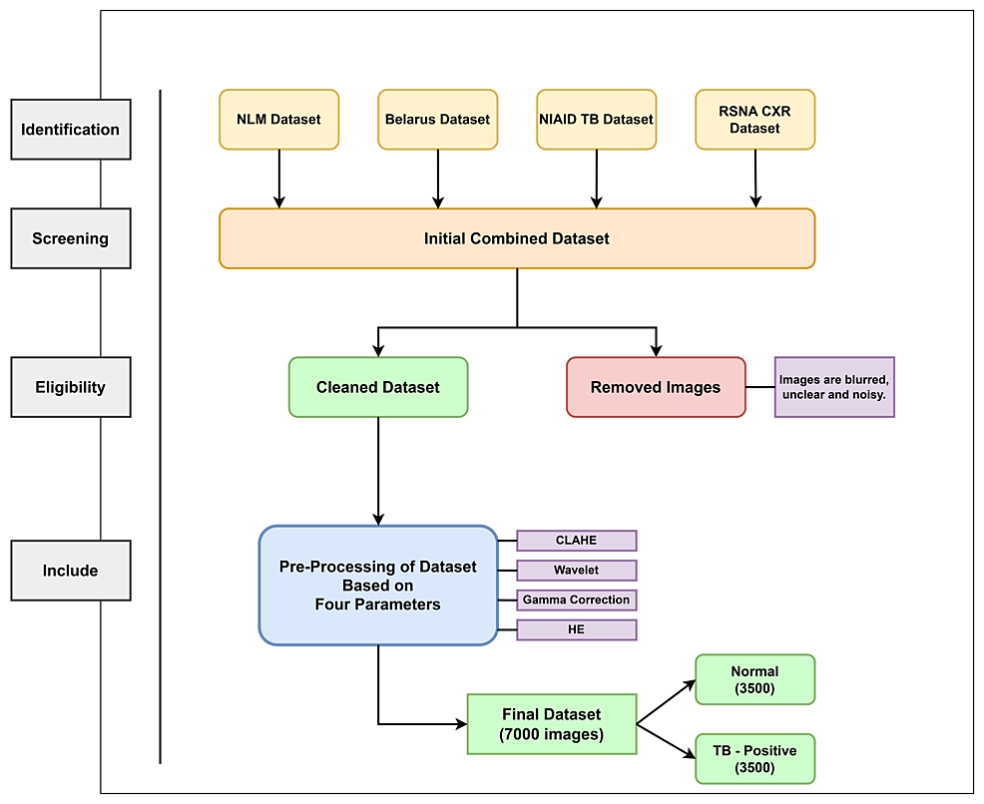
Visual representation of pre-processing of the dataset NLM, National Library of Medicine;  CXR, chest X-ray;  NIAID, National Institute of Allergy and Infectious Diseases;  TB, tuberculosis;  RSNA, Radiological Society of North America;  CLAHE, contrast limited adaptive histogram equalisation;  HE, histogram equalisation. CLAHE, Gamma Correction, HE, and Wavelet techniques help to transform images through noise compression, pixel intensity redistribution, and image fusion.

We used four phases for the data preprocessing phase: identification, screening, eligibility, and the final dataset. Chest X-ray pictures were gathered from four important sources for the identification step. We sent all of the records for the second screening stage, which verified the accuracy of the records from the first stage, after the data-gathering process. Next comes the eligibility phase, when we remove any noisy or grainy images that can affect the accuracy of our results. The primary goal was to achieve the qualifying requirements in order to improve the model's TB diagnosis accuracy. Using preprocessing techniques including wavelet transformation, gamma correction, adaptive contrast with limited equalisation of the histogram (CLAHE), and equalisation of the histogram on the stage 3 images that were received was the final step. Each of these methods was essential in getting the dataset ready for further examination and model training. Following every step of the preprocessing procedure, Figure 2 shows how we arrived at 7,000 final pictures, of which 3500 were classified as normal and 3500 as tuberculosis [6].

Data augmentation

In order to save our model from overfitting and to implement generalisation, we used some augmentation techniques on the images, such as shifting, scaling, random rotation, flipping, and brightness controlling. By doing this, our training data set becomes more diverse, which enhances the model's capacity to generalise to previously undiscovered data.

**Figure 3:**
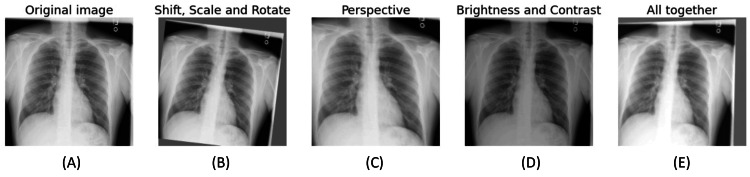
Sample of data augmentation A-E highlights a variety of augmentation techniques applied to the images. A depicts the original image. B shows the changes in rotation and scale. C shows the perspective change, while D highlights the brightness changes. Putting all this together, E displays the final image after applying A-D techniques.

Figure 3 shows a comparison of the original image and further augmentation techniques applied to it.

Convolutional neural networks (CNNs)

Deep learning has attracted a large chunk of attention lately as a powerful technique for handling difficult problems. The area of machine learning was born out of the necessity of incorporating the process, known as learning, into machines. Traditional machine-learning techniques need feature extraction, which is a challenging process that often requires domain expertise. CNNs and other deep learning algorithms automatically detect significant features in raw input data, doing away with the need for feature selection. Processing layers in CNNs recognize different data features by applying different abstraction levels. CNNs are highly helpful in many applications, such as speech recognition, photo identification, and natural language comprehension, due to their ability to develop unique qualities. Weight sharing allows CNNs to train fewer parameters, which enhances generalisation and decreases the likelihood of overfitting. When compared to generic artificial neural network (ANN) models, this integration makes the design of huge networks easier.

**Figure 4:**
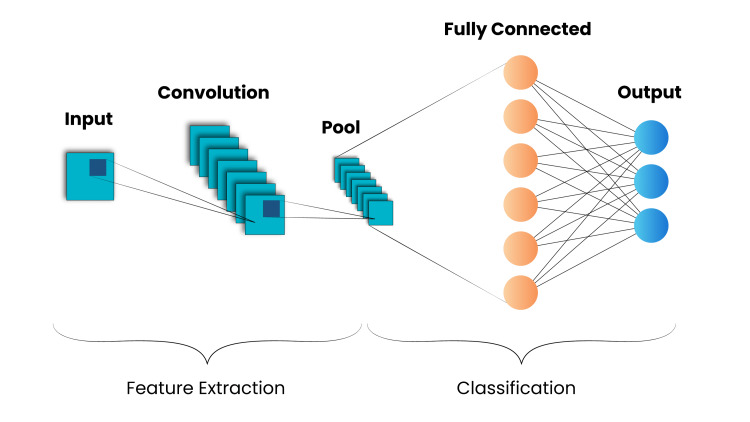
Representation of generalised CNN CNN, convolutional neural network.

Figure 4 represents the generalised idea of how CNN works in two stages: classification and feature extraction using multiple layers.

Components of CNN

Convolutional Layer

The convolution layer uses features extracted from the image to generate the predicted class label, the output, when it receives an image as input. A local relationship known as a receptive field connects every neuron in the next layer to every other neuron in the layer before it. We use these receptive fields to extract local details from the input image. Through the plane of neurons in the next layer, the weight vector associated with a neuron's receptive field does not change. In the convolution layer, this weight vector-also known as a filter or kernel-works on the input vector to produce a feature map. This procedure produces numerous filters and feature maps, generating several features from the original image.

Pooling/Squeezing Layer

After the convolution layer, we use the pooling, or squeezing, layer, primarily to reduce the less important features from various regions. This diminishes the overall number of trainable parameters while adding translation invariance. During the pooling process, we choose a specific shape for the sliding window and then process each feature map from the convolutional layer using a pooling function to produce an output vector. Our model uses max-pooling, which selects the maximum value from the window, due to its effectiveness in squeezing the feature map size.

Completely Networked Layer

The fully connected layer, which gets the output from the phase before it, which consists of pooling and convolution layers, determines the final result by computing the dot product of the input vector and the weight vector. Dense and flatten are the two main layers that are typically present. Multi-dimensional feature maps are produced by the convolutional and pooling layers, and the Flatten layer converts these into one-dimensional vectors [7]. This flattened vector is then given to the Dense layers for classification or regression.

Activation Function

We use the activation function to add non-linearity to the network and improve its accuracy. We have used the Rectified Linear Unit (ReLU) in the convolutional and hidden layers, which is easier to calculate, requires less training time than sigmoid activation functions, and has demonstrated superior performance in machine learning algorithms. As our dataset comprises binary classes, we have employed the sigmoid function to ascertain whether the output is normal or TB-positive.

Optimizers

Deep learning model training requires optimizers since they alter model inputs to reduce the loss determined by different techniques. Through weight changes based on the gradients of the loss function with respect to the weights, they ascertain the learning process of the model. In this study, we utilized the Adam optimizer, known for its modifiable learning rate methodology. Adam may adaptably alter the learning rates during training as a result of this feature, which makes it effective and helpful for a range of deep learning applications.

Loss Function

In our study, we used the binary cross-entropy loss function. Binary cross-entropy is often used for binary classification tasks, such as the one in our study where we had to distinguish between TB-positive and TB-negative cases. It computes the difference between two probability distributions for binary classification, one reflecting the actual labels and the other the expected probabilities. The goal is to minimise this discrepancy because it shows a better match between the predicted and real labels.

Model architecture diagram

**Figure 5:**
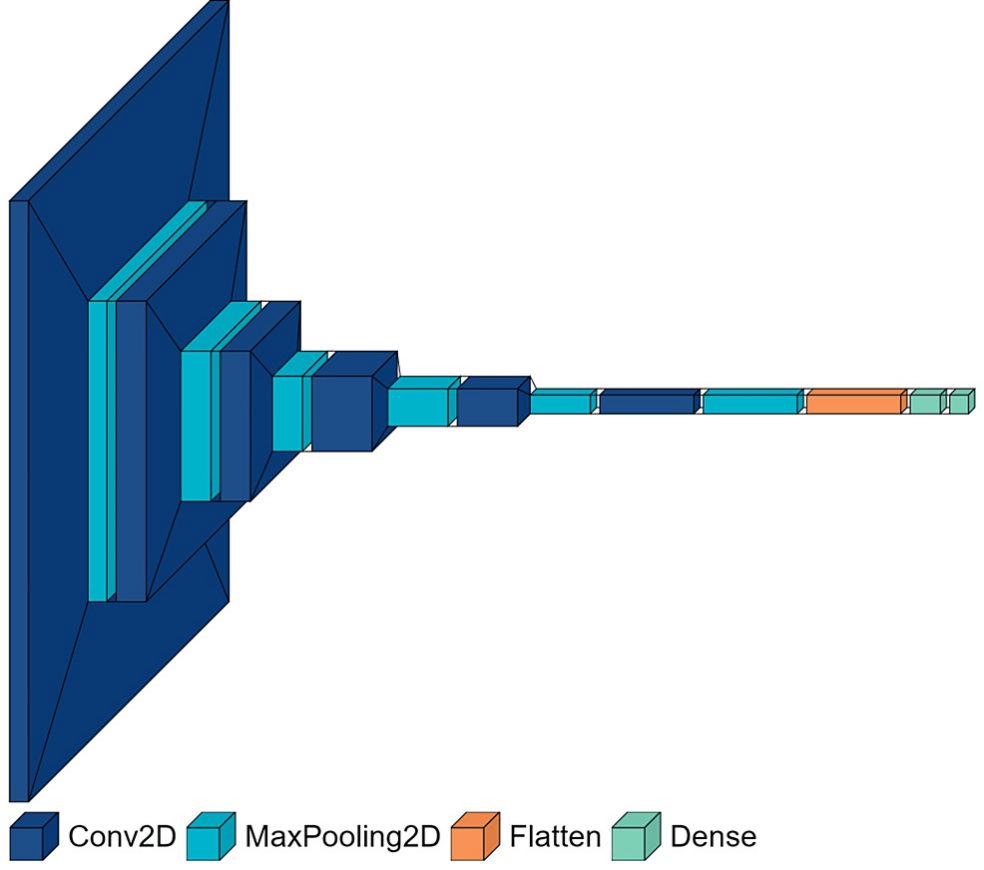
Visual representation of CNN architecture showcasing the different layers of the used model The model comprises of mainly four types of layers: Conv2D represents the convolutional layer, MaxPoolling2D represents the pooling layer, Flatten represents the dimensionality reduction layer, and Dense represents the output layer. CNN, convolutional neural network.

Figure 5 displays the CNN architecture used for our model in this study.

CNNs have drawn interest because of their capacity to classify data depending on context. The primary components of the traditional CNN model are the activation function, pooling layer, convolution layer, optimizers, loss functions, and fully linked layer.

## Results

Model performance

After training our model, we verified its results using our 700-image test set. We determined the model's performance through various metrics: F1-score, receiver operating characteristics (ROC) curve, accuracy, precision, recall, and recall. These results demonstrate the accuracy with which the CNN model identified tuberculosis in chest X-ray images.

Confusion Matrix

**Figure 6:**
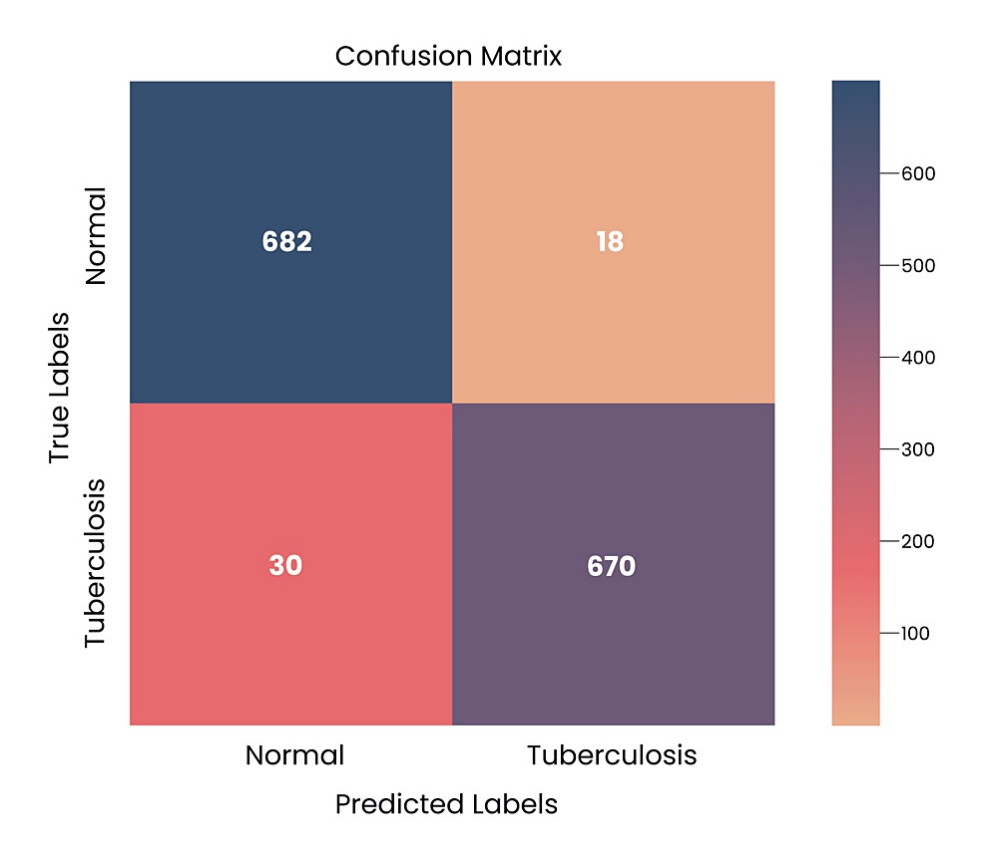
Confusion matrix illustrating the suggested model's performance over test dataset of 700 images The overall accuracy of the model comes out to be ~(96.57) %

It shows how well the model predicts in comparison to the actual labels on the chest X-rays. Figure 6 shows the confusion matrix for the test dataset. 

Score for Accuracy

We define accuracy as how many correct results the model predicts in comparison to all the actual inputs. We achieved an impressive ~(96.57) % accuracy rate on our testing dataset. We tracked the CNN model's training development throughout 50 epochs. The model was successfully learning the properties of the data, as evidenced by the steady decrease in its loss.

 Accuracy = (TP + TN) / (FP + TN + TP + FN)

 TP = True Positive, TN = True Negative, FP = False Positive, FN = False Negative

**Figure 7:**
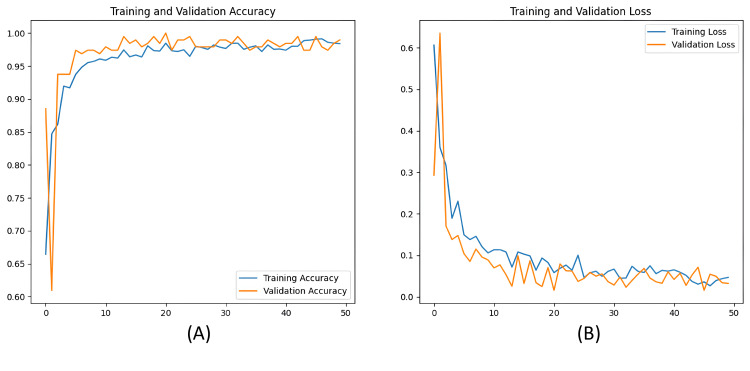
Graphical depiction of the model's accuracy and loss across the number of epochs A-B highlights the accuracy and loss over the increasing epochs. A shows the training and validation accuracy over 50 epochs, while B shows the training and validation loss.

Figure 7 below depicts the validation accuracy and validation loss against epochs.

Score for Precision

Precision can be defined as how accurately our model predicts results in comparison to the actual true results. We finished after reaching a 97% average precision score.

 Precision = TP / (FP + TP)

Score for Recall (Sensitivity)

It is the comparison between all observations made in the actual class and all positively predicted labels. We achieved a 96% sensitivity score.

 Recall = TP / (TP + FN) 

Precision-Recall Curve

**Figure 8:**
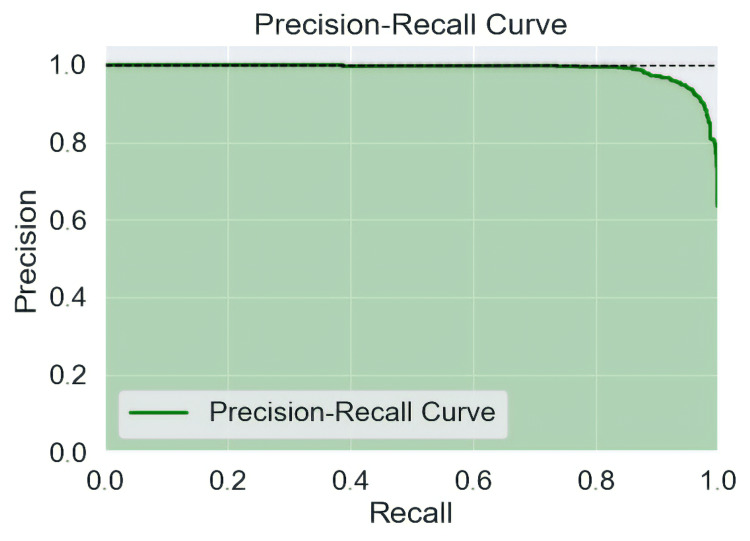
The link between recall and precision throughout the number of epochs is represented by a curve.

High precision and high recall are shown by a high area under the curve, which depicts the model's excellent performance. Figure 8 demonstrates the precision-recall curve*.*

*ROC Curve*

**Figure 9:**
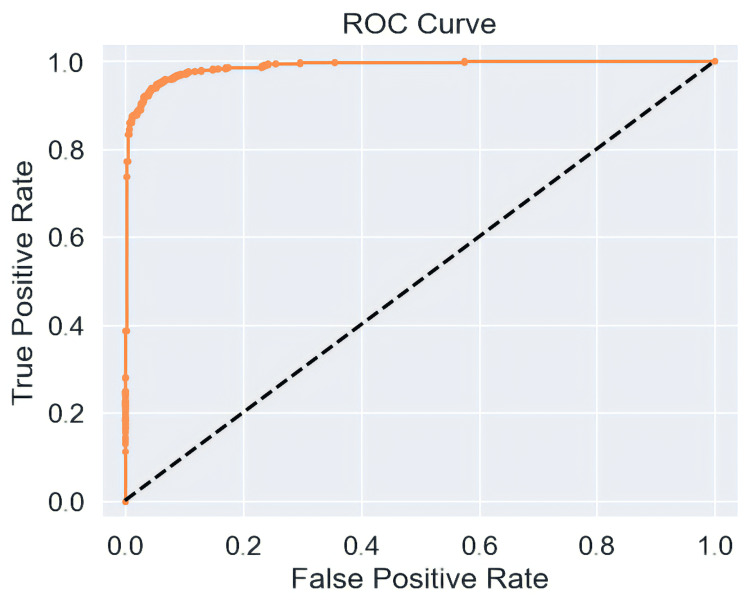
ROC curve illustrates the relationship between True Positive derivative and False Positive derivative over the number of epochs ROC, Receiver Operating Characteristic

The ROC curve, a graphical representation, illustrates how well a binary classifier system can identify issues as its discrimination threshold is adjusted. Figure 9 below represents the ROC curve.

F1 Score

It is the weighted average between precision and recall. After getting the results, we attained an F1 score of ~96%.

 F1 score = 2 * (Precision * Recall) / (Precision + Recall)

## Discussion

The results of the study show how deep learning can be significantly beneficial for tuberculosis detection. CXR imaging (images of chest x-rays) turns out to be an affordable and practical method for medical practitioners without much human burden for diagnosing tuberculosis (TB). Using a cleaned dataset of 7,000 chest X-ray images, containing two labels: TB-positive and normal individuals, we demonstrated a model that showed excellent results without using pre-trained models, which require a lot of processing power and computation.

The scores for both the ROC AUC and the precision-recall AUC were nearly ~99%. This elaborates on the fact that the model properly identified a significant portion of actual TB-positive cases. The model's ability to correctly identify normal situations and avoid making inaccurate predictions is proven by the peak validation accuracy, which reached approximately ~100% in several epochs. These results tend to prove that independently trained models without pre-trained weights can also generate significant accuracy for the diagnosis of tuberculosis (TB).

In this work, we investigated the ability of a separate-training Convolutional Neural Network (CNN) architecture with less convolution and max-pooling layers for tuberculosis symptoms recognition in X-ray images. Our key premise is that a minimum collection of convolutional or feature extraction layers may efficiently handle basic picture classification tasks, like developing a tuberculosis detection system. Interestingly, a large percentage of the research that has already been written in this field tends to focus on deeper convolutional neural networks, especially pre-trained models that demand a lot of processing power [8].

Comparison with current techniques

Our CNN model performs better than current techniques for diagnosing tuberculosis. Previous techniques have used pre-trained models using transfer learning which demands high computation resources. Our model demonstrated an improvement of accuracy with little computational power over various methods used in the past, demonstrating the promise of deep learning approaches for tuberculosis detection.

When we compared our model's performance in this study to that of various other pre-trained models, accuracy was found to be the best. Previous literature has primarily used the Montgomery and Shenzhen datasets as their primary source of data due to their high-quality images, which require high computation power. The average AUC of our model was found to be ~99%, which shows the capability of the model in identifying TB-positive cases from CXRs.

Comparison Table

Table 1 shows the comparison based on several parameters between the pre-trained models to our independently trained model.

**Table 1:** Comparing the suggested approach to earlier literature techniques AUC, area under curve; CNN, convolutional neural network.

Author (year)	Methods	Dataset (image size)	Total number of images used (TB = Normal)	Accuracy (%)	AUC
Chia-Jung Liu et al. (2023). [9]	DenseNet	MIMIC + CheXpert	1500 (780 + 720)	66.5	81.3
Ahmed et al. (2023). [10]	Topo-CXR	Shenzhen CXR	662 (326 + 336)	89.5	93.6
Ahsan et al. (2019). [11]	VGG16	Montgomery + Shenzhen	800 (394 + 406)	81.25	NA
Devasia et al. (2022). [12]	ResNet50	Shenzhen + Montgomery County	3040	76.8	NA
Rajaraman et al. (2021). [13]	ResNet-BS	Montgomery + Shenzhen	800 (394 + 406)	92.30	96
Pattanasuwan et al. (2021). [14]	DenseNet	Montgomery, Shenzhen, and Bureau of tuberculosis	NA	91	95
Nijiati et al. (2021). [15]	TB-UNet	Local CXR data	2903 + 7994	85	NA
Present Work	Self-Trained CNN	Tuberculosis (TB) Chest X-ray Cleaned Database	7000 (3500 + 3500)	96.57	0.99

Integration with clinical practice

There could be a big influence on clinical practice if deep learning models, like CNNs, are used in TB diagnosis, especially in economically backward areas. These models can help radiologists diagnose tuberculosis (TB) more quickly and accurately by helping them analyse chest X-ray pictures, which could help in the early prevention of this infectious disease. Furthermore, CNNs are useful tools for screening programmes and epidemiological investigations because of their speedy analysis of vast datasets.

Limitations

The study's conclusions would undoubtedly be strengthened by increasing the dataset's sample size. However, it's important to remember that training a huge dataset necessitates a lot of computational power and resources, which smaller healthcare facilities might not have on hand. This restriction highlights the need for cooperation and shared resource access in order to overcome such obstacles in subsequent research projects. It is true that despite this level of accuracy, many medical facilities still favour manual TB testing since it raises concerns about a patient's life or death. Still, these techniques save a significant amount of time when compared to manual testing. Research on these subjects is always expanding, and eventually, it may be incorporated into actual situations.

Future scope

In order to enhance the model's performance, we intend to incorporate a variety of datasets, mostly from nearby hospitals that are at risk for tuberculosis. By employing machine learning on CXRs, we intend to raise knowledge of tuberculosis diagnosis among those who are not medical professionals. By adding more workstations and strong graphics processing units (GPUs), we want to explore the usage of various pre-trained CNN models for feature extraction as well as the mixing of multiple network layers.

## Conclusions

Our study features a CNN independently trained to detect TB from chest X-rays (CXRs). To make sure the necessary portion of the original image was present, we submitted both the original and segmented photos. Next, we deployed the CNN model to gain various insights about various features from every image. Training the model with no pre-trained weights required fewer processing resources and yielded a respectable accuracy of ~96.57%. Visualisation metrics, including the ROC curve, precision recall curve, validation accuracy, and loss curve, were used to analyse how well the model performed. The advanced performance of this study can be a quick diagnostic tool, greatly lowering the yearly mortality toll from incorrect or delayed diagnosis.
